# Early exposure to added sugars via infant formula may explain high intakes of added sugars during complementary feeding beyond maternal modeling

**DOI:** 10.3389/fnut.2023.1188852

**Published:** 2023-09-07

**Authors:** Adrianne K. Griebel-Thompson, Tera L. Fazzino, Emily Cramer, Rocco A. Paluch, Katherine S. Morris, Kai Ling Kong

**Affiliations:** ^1^Baby Health Behavior Lab, Division of Health Services and Outcomes Research, Children’s Mercy Hospital, Kansas City, MO, United States; ^2^Department of Psychology, University of Kansas, Lawrence, KS, United States; ^3^Cofrin Logan Center for Addiction Research and Treatment, University of Kansas, Lawrence, KS, United States; ^4^Biostatistics and Epidemiology Core, Division of Health Services and Outcomes Research, Children’s Mercy Hospital, Kansas City, MO, United States; ^5^Department of Pediatrics, University of Missouri-Kansas City, Kansas City, MO, United States; ^6^Division of Behavioral Medicine, Department of Pediatrics, Jacobs School of Medicine and Biomedical Sciences, University at Buffalo, Buffalo, NY, United States; ^7^FeedMore WNY, Buffalo, NY, United States; ^8^Center for Children’s Healthy Lifestyles and Nutrition, University of Kansas Medical Center, Kansas City, KS, United States

**Keywords:** parental modeling, infant formula, added sugar intakes, infant dietary intake, 24-h dietary recall

## Abstract

**Background:**

Research has shown that early exposure to added sugars from table food is related to increased intake of added sugars in later childhood. The earliest window of exposure to added sugars may be in infancy via infant formula. However, beyond the well-established factors of maternal lifestyle and modeling, there is a lack of research examining how exposure to added sugars from infant formula influences infant/toddler added sugar intakes from table foods and sugar sweetened beverages (SSB).

**Objective:**

While accounting factors previously associated with infant/toddler added sugar intakes and maternal SSB consumption (proximal measure of maternal modeling), this study aims to examine if there is an association between added sugars in infant formula and added sugar intakes from table foods and SSB during the complementary feeding period.

**Methods:**

This is a secondary, cross-sectional analysis using three-day caregiver-reported 24-h dietary recalls in a cohort of infant/toddlers (*n* = 95), ages 9- < 16 mos., enrolled in a music intervention trial. Hierarchical stepwise regression was used to estimate the association between exposure to added sugars from infant formula and (1) intake of added sugars from table food and (2) SSB consumption. Infant/toddler SSB consumption was transformed to account for distributional properties. We performed incremental F-tests to determine whether the addition of each step improved model fit (*R*^2^).

**Results:**

Early exposure to added sugars via infant formula was associated with infant/toddler SSB (Δ*R*^2^ = 0.044, Finc (1, 87) =6.009, *p* = 0.016) beyond sociodemographic and maternal SSB consumption, but not with infant/toddler added sugar intakes from table foods (Δ*R*^2^ = 0.02, Finc (1, 87) =3.308, *p* = 0.072).

**Conclusion:**

While past studies have identified circumstantial (i.e., sociodemographic), or indirect (i.e., maternal lifestyle and modeling), mechanisms contributing to higher infant/toddler added sugar intakes, this study identifies exposure to added sugars from infant formula as a possible direct mechanism explaining why some infants/toddlers consume more added sugars.

## 1. Introduction

Despite recent preventative efforts, childhood obesity remains a prevalent health concern for children in the United States. Among the dietary factors related to childhood obesity risk, added sugars have emerged as a leading factor that contributes to the development of childhood obesity (1, 2). As a result, the Dietary Guidelines for Americans (3) and the American Heart Association (4) recommend that children under the age of 2 years avoid added sugars. Alongside obesity (1, 2), the intake of added sugars during infancy and toddlerhood has been associated with early rapid weight gain (5), dental caries (6), asthma (7), cardiovascular disease (4), elevated blood pressure (8), and elevated triglycerides (8).

One well-established factor associated with added sugar intakes is maternal lifestyle and behavioral modeling in relation to dietary intake. Several studies have demonstrated the association of maternal diet on children’s dietary preference. For instance, Scaglioni et al. reported that parental food habits are suggested to influence child food choices (9). Vepsäläinen et al. (10) found that a child’s diet most closely resembles the diet of whomever provides the food to the child, and that is typically the mother (11). Ha et al. (11) and Brekke et al. (12) specifically investigated child added sugar intakes in relation to maternal dietary habits, and they found that low fruit intake and the consumption of sweets during pregnancy was associated with child added sugar intakes. Furthermore, the study of Australian mother-infant dyads, conducted by Ha et al. reported that infants with mothers who consume sugar sweetened beverages (SSB) are 1.8 times more likely to consume added sugars in comparison to children whose mothers did not consume SSB (11). Our team’s recent publication further concurred the findings of Ha et al. supporting that maternal SSB intake is significantly associated with infant/toddler added sugar intakes (13).

Despite a substantial amount of literature relating maternal lifestyle and dietary modeling to infant/toddler added sugar intakes, this factor does not explain the direct mechanism leading to the high added sugar intakes. One possible understudied mechanism affecting infant/toddler added sugar intakes may be early exposure to added sugars via early feeding practices (i.e., infant formula feeding, breastfeeding, or mixture of the two) (14). Research has shown that early exposure to added sugars from table food is related to increased intake of added sugars in later childhood (15); however, this may not be the earliest exposure window to added sugars for most infants. The first nutrient source infants are exposed to is milk, either in the form of breastmilk or infant formula. Although the nutrient profile of infant formula is advertised to be similar to breastmilk, our recent research identified that there are substantial differences between the two sources (14, 16). Our study revealed that most infant formulas produced in the US contain added sugars up to 7.7 g/100 kcal serving (14, 16). As such, when formula is consumed exclusively, infants may yield a daily consumption of added sugars equivalent to two soft drinks (14, 16). Thus, exposure to added sugars through infant formula may be extensive in the first year of life, and particularly during the first 6 months.

Recently, our team proposed an application of the exposure-learning paradigm in order to explain how early exposure to added sugars via infant formula may lead to a greater preference for sweet tastes and therefore the consumption of more added sugars (14). Furthermore, the sugars (e.g., glucose and corn syrup) commonly found in infant formula are known to provoke a sense of pleasure. These sugars stimulate the brain’s reward system into releasing a greater amount of dopamine, reenforcing the consumption of added sugars (14). Research has shown that consumption of toddler formula, which typically contains high amounts of added sugars [10.5 g/8 fl. oz. serving calculated using Nutrition Data System for Research (NDSR)], is correlated to increased added sugar intakes later in childhood (17, 18). However, there are no studies to our knowledge that have examined the consumption of infant formula at the earliest possible window, which is during infancy. In this study, we examined whether exposure to added sugar in infant formula explains the variance in US infant/toddler consuming formula manufactured in the US (1) added sugar intakes from table foods (i.e., all other dietary sources outside of infant formula) and (2) SSB consumption at 9- < 16 mos., beyond the well-established factors such as sociodemographic and maternal lifestyles and dietary modeling. We hypothesize that exposure to added sugars via infant formula during infancy is associated with infant/toddler added sugar intakes from table foods and SSB consumption at 9- < 16 mos. We hypothesize these results will remain present despite accounting for sociodemographic and maternal modeling behavior. In our analysis, maternal SSB consumption will serve as the proxy measure of maternal lifestyles and behavioral modeling per our previous work, Griebel-Thompson et al. (13) and the work of Ha et al. (11).

## 2. Materials and methods

### 2.1. Participants

**Figure 1:**
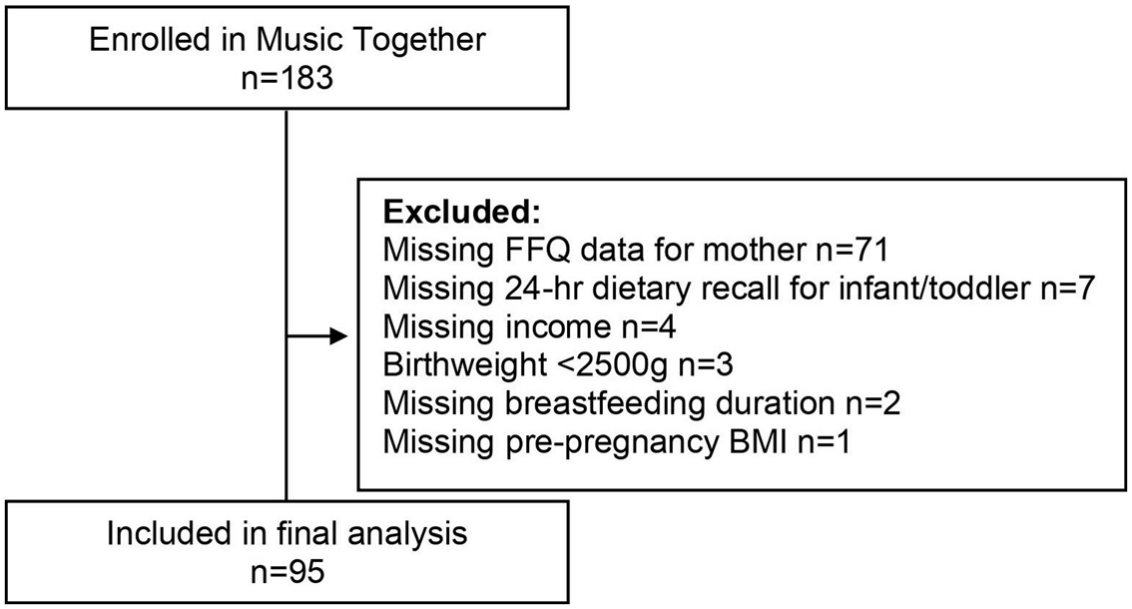
Participant flow chart.

This cross-sectional analysis included baseline data from 95 mother-infant/toddler dyads, age 9- < 16 mos., (Figure 1) participating in a longitudinal randomized controlled trial (ClinicalTrials.gov Identifier: NCT02936284) (5). Trained study personnel obtained informed consent from parents of eligible participants. Participants were able to withdraw at any point of this study. Exclusion criteria included: infants born preterm (<37 weeks gestation), low-birth weight infants (<2,500 g), infants/toddlers with known medical problems, infants/toddlers consuming special diets, infants/toddlers with developmental delays or disabilities, maternal smoking, alcohol abuse, or controlled substance use during pregnancy, mother <18 years of age, high risk pregnancy (Gestational Diabetes Mellitus, pre-eclampsia, etc.), or multiple gestation (5). In accordance with the Institute of Medicine’s Food and Nutrition Board (19), two participants who consumed calories estimated to be ±2 SD from their estimated energy requirement were not included in the present analysis. The University at Buffalo Institutional Review Board (IRB) approved this study (protocol code STUDY00000472 and 5/24/2016).

### 2.2. Demographic and pregnancy history and feeding practices questionnaire

Researchers applied the shortened form of the feeding questionnaire from the Infant Feeding Practices Study II (20) in order to collect information for this study. The short form of the Infant Feeding Practices Study II uses 13 questions which assess areas of feeding practices including, but not limited to, the initiation and duration of breastfeeding, timing of solid food introduction, age of infant/toddler at cessation of breastfeeding, and maternal pregnancy history. The questionnaire was administered online using SurveyMonkey (https://www.surveymonkey.com, accessed on 24 May 2016).

### 2.3. Dietary intake

#### 2.3.1. Maternal dietary intake

The application of the Block 2014 Food and Activity Questionnaire, developed by NutritionQuest (Berkley, CA, USA) (21), gathered information on maternal dietary intake. The Block 2014 is a full-length food frequency questionnaire (FFQ) comprised of 127 food and beverage items. It utilizes questions that specifically measure fat, carbohydrate, sugar, and whole grain intakes (21). The 2007–2008 and 2009–2010 NHANES cycles were used to design the Block 2014, while the USDA’s Food and Nutrient Database for Dietary Studies (FNDDS 5.0), Food Pyramid Equivalents Database (FPED), and Nutrient Database for Standard Reference (SR27) were used to create the nutrient and food group analysis database (21). This FFQ assesses SSB intake of sugary beverages including fruit juice, soda, and energy drinks (21). Respondents are provided with options to select from for frequency of intake including: (1) every day, (2) 5–6 times per week, (3) 3–4 times per week, (4) 2 times per week, (5) once per week, (6) 2–3 times per month, (7) once per month, (8) a few times per year, and (9) never.

#### 2.3.2. Infant or toddler dietary intake

Infant/toddler dietary intake, including intake of added sugars from infant formula (i.e., sugars added above the level of naturally occurring lactose in the milk-base used in infant formula manufacturing and other added sugars, such as corn syrup solids or glucose), was assessed by three caregiver-reported 24-h dietary recalls. As conducted in the Feeding Infants and Toddlers Study (FITS), trained study personnel collected 24-h dietary recalls from parents/caregivers by phone on random occasions (two weekday, one weekend day) within 10 days of anthropometric measurement (22). We provided parents with handouts in order to ensure accurate reporting. These handouts contained tips on how to report serving sizes, along with answers to frequently asked questions (5). Furthermore, study personnel, trained by a registered dietitian (MS/RD), collected information from parents regarding infant/toddler feeding. If parents reported that the infant/toddler did not have a normal eating day within the past 24-h period, then the recall was administered on a different day (5, 22). These 24-h dietary recalls were derive from the USDA Automated Multiple-Pass Method (23). All study personnel used a script to administer 24-dietary recalls in order to ensure adherence to protocol. We did not include the intake of medicine or supplements in this analysis. Kong et al. described this methodology previously (5).

Information regarding breastfeeding was obtained based on parents’ records of both the duration at the breast and the intake of human milk (5, 22). Reports on nutritional intake from human milk follow the methodology described by the FITS study (22). Briefly, for exclusively breastfed infants ages 7–12 mos, 600 mL of human milk was reported (5, 22). For those consuming both human milk and infant formula, the amount of formula reported was subtracted from 600 mL, and the remainder was reported as human milk (5, 22). For toddlers ≥12 mos., 1 fl. oz. of human milk was reported for every 5 min at the breast (5, 22). We also collected detailed information on the brand of formula consumed including preparation methods and amounts consumed. Finally, SSB are any sugary beverages excluding milk and infant formula/human milk.

Infant dietary intake data, including infant formula brand and types, were collected and analyzed using NDSR software version 2019, which was developed by the Nutrition Coordinating Center (NCC), University of Minnesota, Minneapolis, MN (24–26). Study personnel selected generic food in substitute of specific food that could not be found in the database. A total of 15 foods without a comparable generic food were not in the database. The NCC was contacted, and the foods were added to the database.

### 2.4. Statistical analysis

Participant demographic information is reported in either mean and standard deviation or sample size and percentage. Infant/toddler SSB consumption was log transformed to account for distributional properties. Hierarchal stepwise regression estimated whether added sugar intakes from formula (kcal) at 9- < 16 mos. Was associated with infant/toddler added sugar intakes from table foods (kcal) or SSB at 9- < 16 mos., beyond established predictors related to material dietary modeling. In model 1, we controlled for demographic characteristics reported to be associated with infant/toddler added sugar intakes. These include infant/toddler sex, infant/toddler age (mos.), birthweight (kg), parity, maternal body mass index (BMI) (kg/m^2^), maternal education (y), family income, first introduction of solid foods (mos.), and breastfeeding duration (mos.). In model 2, we controlled for our previously published (13) proxy of maternal modeling, maternal SSB consumption. In model 3, we included added sugars from infant formula (kcal). Incremental F-tests determined whether there was any occurrence of statistically significant change by comparing improvements in the model fit (*R*^2^). We also report the effect size of the *F* statistic which is a measure of the strength of relationship between two variables. The variance inflation factor (VIF) was calculated in all models, and multicollinearity was not observed. Statistical analysis was performed using SAS 9.4 (SAS Institute Inc., Cary, NC, United States, 2020) (27).

## 3. Results

Table 1 presents demographic information of mothers and infants/toddlers. Table 2 shows the results of the hierarchical stepwise regression models depicting the association between exposure to added sugars via infant formula and the outcome of infant/toddler added sugar intakes from table foods. In step 1, 43.9% of variance was accounted for by demographic characteristics that were reported in prior literature to be associated with infant/toddler added sugar intakes. By adding maternal SSB intakes (a proxy maternal modeling) in step 2, the total variance increased to 45.4%. However maternal SSB intakes were not significantly associated with infant/toddler added sugar intakes from table foods (Δ*R*^2^ = 0.015, Finc (1, 88) = 2.418, *p* = 0.124). Model 3 accounts for exposure to added sugars via infant formula. The total variance increased to 47.4%, and early exposure of added sugars via infant formula was not significantly associated with infant/toddler added sugar intakes from table foods (Δ*R*^2^ = 0.02, Finc (1, 87) =3.308, *p* = 0.072).

**Table 1:** Participant characteristics (*n* = 95).

	Mean (SD)	Range	*N* (%)
*Child*			
Sex, male			42 (44.2)
Race, Caucasian			81 (85.3)
Age, mos.	11.8 (1.8)	9.1–15.8	
Gestational age, weeks	39.4 (1.1)	37–42	
Birth weight, kg	3.5 (0.5)	2.6–5.2	
Weight-for-length z-score^a^	0.56 (0.84)	−1.3-3.1	
Weight-for-age z-score^a^	0.20 (0.9)	−2.4-2.6	
Length-for-age z-score^a^	−0.3 (1.2)	−3.1-2.9	
Conditional weight gain^b^	0.03 (0/97)	−2.8-2.3	
Breastfeeding duration			
< 6 mos.			26 (27.4)
≥ 6 mos.			69 (72.6)
First introduction to solid foods			
< 4 mos.			3 (3.2)
4–5 mos.			38 (40.0)
≥ 6 mos.			54 (56.8)
Intake of infant formula (kcal)	115.7 (188.2)	0–637.7	
Intake of added sugars from infant formula (kcal)	26.0 (44.4)	0–151.8	
Intake of sugar-sweetened beverages (kcal)	34.8 (108.8)	0–720	
Intake of added sugar from table foods (kcal)	25.8 (22.5)	0–100.9	
Total energy intake (kcal)	911.0 (214.2)	483–1,445	
*Mother*			
Age, y	32.4 (4.1)	22–45	
Education level			
Some college or below			15 (15.8)
College graduate or higher			80 (84.2)
Number of children			
1 child			54 (56.8)
≥ 1 child			41 (43.2)
Current BMI, kg/m^2^			
Normal weight			31 (32.6)
Overweight/obese (≥25 BMI)			64 (67.4)
Household total income			
< $30,000			0 (0.0)
$30,000 - $69,999			28 (29.5)
$70,000 - $109,999			41 (43.2)
≥ $110,000			26 (27.4)

a Calculated by using the WHO growth charts.

b Calculated using method described by Griffiths et al. (2008) (29).

SD, Standard Deviation.

**Table 2:** Hierarchical regression models of added sugar intakes from formula [*R^2^*, change statistics (*ΔR^2^*), and regression coefficients (*β*)] predicting infant added sugar from table food intake (*n* = 95).

Effect	*R*^2^	*ΔR^2^*	*β*	*t*	*p*-value	Effect size	95% Confidence interval of the effect size	
*Step 1*								
Child sex			−3.169	−0.82	0.415	0.0078	0.000	0.075
Child age (mos.)			7.272	6.78	<0.0001^*^	0.3508	0.179	0.459
Birth weight (kg)			−3.983	−0.97	0.336	0.0109	0.000	0.083
Parity			0.897	0.43	0.670	0.0022	0.000	0.055
Maternal BMI (kg/m^2^)			−0.023	−0.08	0.935	0.0001	0.000	0.020
Maternal education (y)			−2.672	−2.60	0.011^*^	0.0737	0.004	0.182
Family income			0.347	0.30	0.768	0.0010	0.000	0.046
First introduction to solid foods (mos.)			−0.577	−0.26	0.798	0.0008	0.000	0.043
Breastfeeding duration (mos.)			−0.816	−1.67	0.098	0.0319	0.000	0.123
	0.439							
*Step 2*								
Maternal sugar sweetened beverage consumption			0.011	1.53	0.131	0.027	0.000	0.114
Finc (1, 88) = 2.418, *p* = 0.124	0.454	0.015						
*Step 3*								
Added sugars from infant formula (kcal)			−0.092	−1.79	0.077	0.037	0.000	0.130
Finc (1, 87) = 3.308, *p* = 0.072	0.474	0.02						

^*^ Indicates *p* < 0.05.

Table 3 displays the results of the hierarchical stepwise regression models regarding the association between exposure to added sugars via infant formula and infant/toddler SSB consumption as the outcome. Step 1, which controlled for demographic variables associated with infant/toddler added sugars intake, accounted for 31.3% of variance. The addition of maternal SSB consumption in step 2 increased the amount of variance accounted for to 31.9% (Δ*R*^2^ = 0.006, Finc (1, 88) = 0.775, *p* = 0.381). Maternal SSB consumption was, however, not significantly associated with infant SSB intake. Finally, the addition of exposure to added sugars via infant formula in step 3 accounted for 36.3% of the variance, and the exposure to added sugar via infant formula was significantly associated with infant/toddler SSB consumption (Δ*R*^2^ = 0.044, Finc (1, 87) =6.009, *p* = 0.016*).

**Table 3:** Hierarchical regression models of added sugar intakes from formula [*R^2^*, change statistics (*ΔR^2^*), and regression coefficients (*β*)] predicting infant sugar sweetened beverage intake (*n* = 95).

Effect	*R*^2^	*ΔR^2^*	*β*	*expβ*	*t*	*p*-value	Effect size	95% Confidence interval of the effect size	
*Step 1*									
Child sex			−0.949	0.387	−0.82	0.415	0.0078	0.000	0.075
Child age (mos.)			0.558	1.747	1.74	0.086	0.0343	0.000	0.127
Birth weight (kg)			−2.714	0.066	−2.20	0.030^*^	0.0540	0.000	0.156
Parity			0.696	2.006	1.11	0.270	0.0143	0.000	0.090
Maternal BMI (kg/m^2^)			0.138	1.148	1.62	0.108	0.0301	0.000	0.120
Maternal education (y)			−0.318	0.728	−1.03	0.304	0.0124	0.000	0.086
Family income			−0.658	0.518	−1.87	0.065	0.0396	0.000	0.135
First introduction to solid foods (mos.)			−1.501	0.223	−2.23	0.028^*^	0.0554	0.000	0.158
Breastfeeding duration (mos.)			−0.191	0.826	−1.31	0.195	0.0196	0.000	0.101
	0.313								
*Step 2*									
Maternal sugar sweetened beverage consumption			0.002	1.002	0.88	0.381	0.0092	0.000	0.078
Finc (1, 88) = 0.775, *p* = 0.381	0.319	0.006							
*Step 3*									
Added sugars from infant formula (kcal)			−0.037	0.964	−2.39	0.019^*^	0.0644	0.001	0.168
Finc (1, 87) = 6.009, *p* = 0.016^*^	0.363	0.044							

^*^ Indicates *p* < 0.05.

## 4. Discussion

The intake of added sugars is emerging as a major contributing factor in the development of childhood obesity (1, 2, 13). The earliest possible exposure window to added sugars is via infant formula, but little research has investigated how consumption of infant formula may explain variance in infant added sugar intakes from table foods and SSB within the complementary feeding period. Informed by our recently presented paradigm for the etiology of obesity in early childhood (14), this study examined whether consumption of added sugars in infant formula may be a direct mechanism explaining high intake of added sugar from both table foods and SSB among infants/toddlers during the complementary feeding period. Notably, we examined whether the consumption of added sugars from infant formula explained variance in added sugar intake beyond the factors of sociodemographic and maternal lifestyle and dietary modeling.

Our findings suggest that exposure to added sugars from infant formula is associated with added sugar intakes from SSB of infants/toddlers 9- < 16 mos. of age. While studies investigating this association during infancy are limited, those that have examined it do concur with our findings. Redruello-Requejo et al. (17) studied a cohort of Spanish children and found that those who consumed adapted milks (i.e., toddler formula and enriched milks) in toddlerhood experienced an increase of added sugar intakes with age compared to their counterparts who did not consume adapted milks. Those who did not consume adapted milks experienced more stable levels of added sugar intakes with age. Kostecka et al. (18) studied children ages 13–24 mos. and found that children who drank toddler formula consumed more beverages with sugar including fruit juice, nectars, and sweetened hot beverages (18). While these studies support the premise of our paradigm, that exposure to added sugars from formula may result in an increased consumption of added sugars found in other sources, these studies do not focus on the earliest exposure window (i.e., infant formula during the first year of life). Furthermore, these studies did not consider other potential contributors in their analyses.

Interestingly, the association between early exposure to added sugars through infant formula and intake of added sugars from table foods was not significant in our cohort. While more studies in this area are needed to confirm our result, there are plausible explanations for this finding. First, our sample included infants/toddlers 9- < 16 mos of age who might be consuming little solid food outside of human milk or infant formula. Thus, added sugar intakes from tables foods might be insufficient to estimate its relationship with early exposure to added sugars from infant formula. In the future, a longitudinal study starting at birth may be warranted to pursue this line of work. Next, the fact that our population was rather homogeneous and consisted of highly educated, non-Hispanic/Latino White participants of high-income families may impact this finding. It is well-known that social and demographic factors influence not only the choice to feed infants human milk or infant formula, but also intake of added sugars (28, 30). Finally, it is possible that early exposure to added sugars through infant formula may have a greater influence on SSB intake compared to table food because infant formula is like a beverage, and the exposure learning may be specific to beverages in our present analyses.

In the present study, we recognize that infant/toddler added sugar intakes during the complementary feeding period is complex and multifactorial. Therefore, in our analyses, we first controlled for known or well-established factors such as sociodemographic variables (1, 28), age of introduction of solid foods (31), and lastly maternal SSB consumption (a proxy measure of maternal modeling) in a stepwise manner. To date, the existing literature has only identified circumstantial (i.e., sociodemographic factors) or indirect factors, such as maternal lifestyles and behavioral modeling, as contributors to infant added sugar intakes. Thus, our work may be significant in understanding potential drivers of unhealthful dietary habits. We posit that the consumption of added sugars in infant formula is a direct mechanism, as well as a form of early exposure to artificially reinforcing nutrient sources among children, as described in our recent publication (14). The early exposure to added sugars in infant formula teaches infants that food should be sweet, and this strengthens the inherent preference for sweet foods (e.g., foods high in added sugars) (32–34). This yields to the high intake of added sugars, suggesting it to be a direct mechanism. Furthermore, exposure learning is most influential when there has been no occurrence of prior exposure. This means that infancy may possibly be the most pivotal time for the development of food preference and eating behaviors, making infant formula the most pivotal food source (35, 36).

From a larger, societal perspective, the current food environment makes avoiding added sugars extremely difficult. Popkin et al. found 68% of barcoded foods available in US grocery stores contain added sugars (37). Nearly all the participants in this study consumed added sugars. This demonstrates how difficult it is to follow the recommendation by the Dietary Guidelines for Americans and the American Heart Association that children <2 years avoid added sugars (3, 4). Furthermore, marketing practices in the food industry are problematic. Marketing of infant formula to expecting, new mothers is predatory because not only does it undermine a woman’s right to choose how to feed and care for her infant, but it also undermines public health efforts to promote breastfeeding (38). Similarly, marketing of foods towards children is unethical because children are especially vulnerable to persuasive marketing tactics (39). This is a known contributor to childhood obesity (40). Future research and policy should address both individual and societal level factors in order to reduce the added sugar intakes of children.

A major strength of this study is the use of dietary recall methodology, consisting of three 24-h dietary recalls, which assessed infant intake. Additionally, to ensure accurate reporting, we used the USDA Automated Multiple-Pass Method to collect the 24-h dietary recalls and a registered dietitian (MS/RD) trained all study personnel on this protocol. Application of the hierarchical stepwise regression to statistical analysis strengthened this study. This theory driven technique allowed us to estimate how each step contributed to the overall model. A limitation, however, of this study is likely overestimation from maternal reports on infant/toddler dietary intake (41). It is also a limitation that parenting style (i.e., awareness and perception of the diet-health relationship) and the fact parents provide the food consumed by infants/toddlers 9- < 16 months of age were not accounted for. Lastly, this cohort is predominately white, highly educated, and includes mostly medium and high-income families making results less generalizable, especially to lower income families. Furthermore, the cohort social and demographic homogeneity may explain why previously described variables related to infant/toddler added sugars intake were not significant.

## 5. Conclusion

The current study suggests that early exposure to added sugars via infant formula may play a critical role in added sugar intakes during the complementary feeding period, even with the consideration of established sociodemographic and maternal dietary modeling variables.

## Data availability statement

The raw data supporting the conclusions of this article will be made available by the authors, without undue reservation.

## Ethics statement

The studies involving humans were approved by The University at Buffalo Institutional Review Board (IRB). The studies were conducted in accordance with the local legislation and institutional requirements. Written informed consent for participation in this study was provided by the participants’ legal guardians/next of kin. Informed consent was also obtained from participants’ legal guardians/next of kin for their own participation in the study.

## Author contributions

AG-T, EC, TF, RP, and KK conceptualized the work. KM collected the data. AG-T, RP, and EC analyzed the data. AG-T wrote the first draft with contributions from KK. All authors contributed to the article and approved the submitted version.

## Funding

This work was supported by National Institute on Child Health and Human Development of the National Institutes of Health Grant ID: R01 HD087082.

## Conflict of interest

KM is employed by FeedMore WNY.

The remaining authors declare that the research was conducted in the absence of any commercial or financial relationships that could be construed as a potential conflict of interest.

## Publisher’s note

All claims expressed in this article are solely those of the authors and do not necessarily represent those of their affiliated organizations, or those of the publisher, the editors and the reviewers. Any product that may be evaluated in this article, or claim that may be made by its manufacturer, is not guaranteed or endorsed by the publisher.
